# A study on fractional tumor-immune interaction model related to lung cancer via generalized Laguerre polynomials

**DOI:** 10.1186/s12874-023-02006-3

**Published:** 2023-08-21

**Authors:** Hossein Hassani, Zakieh Avazzadeh, Praveen Agarwal, Samrad Mehrabi, M. J. Ebadi, Mohammad Shafi Dahaghin, Eskandar Naraghirad

**Affiliations:** 1 Department of Mathematics, Anand International College of Engineering, Jaipur, 303012 India; 2 Department of Mathematical Sciences, University of South Africa, Florida, South Africa; 3 Department of Internal Medicine, Shiraz University of Medical Sciences, Shiraz, Iran; 4 Department of Mathematics, Chabahar Maritime University, Chabahar, Iran; 5 Faculty of Mathematics, Shahrekord University, Shahrekord, Iran; 6 Department of Mathematics, Yasouj University, Yasouj, Iran

**Keywords:** Fractional Tumor-Immune Interaction Model, Tumor Cells, Lung Cancer, Generalized Laguerre Polynomials, Macrophages Cells, 92C42, 41A58, 97M60

## Abstract

**Background:**

Cancer, a complex and deadly health concern today, is characterized by forming potentially malignant tumors or cancer cells. The dynamic interaction between these cells and their environment is crucial to the disease. Mathematical models can enhance our understanding of these interactions, helping us predict disease progression and treatment strategies.

**Methods:**

In this study, we develop a fractional tumor-immune interaction model specifically for lung cancer (FTIIM-LC). We present some definitions and significant results related to the Caputo operator. We employ the generalized Laguerre polynomials (GLPs) method to find the optimal solution for the FTIIM-LC model. We then conduct a numerical simulation and compare the results of our method with other techniques and real-world data.

**Results:**

We propose a FTIIM-LC model in this paper. The approximate solution for the proposed model is derived using a series of expansions in a new set of polynomials, the GLPs. To streamline the process, we integrate Lagrange multipliers, GLPs, and operational matrices of fractional and ordinary derivatives. We conduct a numerical simulation to study the effects of varying fractional orders and achieve the expected theoretical results.

**Conclusion:**

The findings of this study demonstrate that the optimization methods used can effectively predict and analyze complex phenomena. This innovative approach can also be applied to other nonlinear differential equations, such as the fractional Klein–Gordon equation, fractional diffusion-wave equation, breast cancer model, and fractional optimal control problems.

## Introduction

Lung cancer is diagnosed as the most common cancer [1, 2]. In 2018, an estimated 2.1 million new lung cancer cases were made, accounting for 12% of the global burden of cancer [1, 2]. Lung cancer is the leading cause of cancer-related fatalities for men while coming in second for women across the globe [1, 2]. Tobacco smoking is in the top spot on the list of risk factors for lung cancer, accounting for 75% of lung cancers. Genetic susceptibility, occupational workplace exposure, air pollution, second-hand smoke, and radon exposure are other risk factors [3].

As of late, there has been a modest enhancement in the survival rate of individuals diagnosed with lung cancer. However, there have been considerable improvements in the chance of survival for most other types of cancer. This can be attributed to most cancer patients being diagnosed at the last stages of the disease [1]. The survival rate for all cancer types diagnosed in 5 years from 2010 to 2016 was 63% among Black individuals, 68% among White individuals, and 67% overall. Lung cancer showed one of the lowest survival rates at about 21% [4]. According to an extensive examination of individuals with lung cancer [5], a substantial percentage of patients, ranging from 40 to 85%, experience respiratory symptoms such as coughing, shortness of breath, wheezing, and coughing up blood. Thus, continuous auscultation and lung sound monitoring [6] can be helpful for early diagnosing of such lung problems.

Lung cancer tumors are categorized into two Histologic Diagnosis groups: Small-Cell Lung Carcinoma (SCLC) and Non-Small Cell Lung Carcinoma (NSCLC). NSCLC accounts for approximately 80 to 85% of all lung cancers, which include subcategories of adenocarcinoma (40%), squamous cell carcinoma (25–30%), and large cell carcinoma (10–15%) [7–9].

The characteristics of its microenvironment influence the progression and dissemination of a tumor. The tumor microenvironment comprises various cell types, including immune cells, cancer cells, vascular endothelial cells, epithelial cells, dendritic cells, macrophages, lymphocytes, fibroblasts, and extracellular matrix proteins. In the respiratory system, the airway epithelium serves as a protective barrier and provides an environment for the growth of lung cancer cells. The epithelial cells release inflammatory mediators that attract lymphoid cells to the airway epithelia and activate antigen-presenting cells (APCs). [10].

The human immune system can search out, detect, and destroy infected or malignant cells while keeping the host safe. However, tumors can potentially evade and escape immune examination and destruction. This escape of tumor cells from immunity includes the local development of immune suppression, induction of dysfunctional T-cell signaling for excessive immune responses, and immune upregulation of deterrent checkpoints against the indiscriminate attack on self-cells [11]. In the anticancer battle, the human body benefits from an arsenal of cytotoxic lymphocytes, macrophages, and granulocytes secreted from immune cells. The Cytotoxic T Lymphocytes (CTLs) population is leading in anticancer immunity. The CD8^+^ lymphocytes, CD4^+^ lymphocytes and lymphocytes B are soldiers of the CTL army, and natural killer (N.K.) cells and natural killer T (NKT) cells are members of the CTL cell population. A successful cytotoxic attack entails an efficient tumor antigen presentation and appropriate antigen-presenting cells (APCs) [12]. Both cytotoxic innate and adaptive immune cells are crucial for anticancer immunity. The innate immune response comprises granulocytes, macrophages, natural killer (NK) cells, mast cells, and dendritic cells (D.C.s). On the other hand, the adaptive response is comprised of B cells, CD8^+^ cytotoxic lymphocytes (CTLs), and CD4^+^ helper T cells [13].

After facing tumor antigens, immature CD4^+^ T cells are made active and polarized. They are divided into Th1, Th2, Th17, Th9, Th22, Tregs, and T follicular helper (Tfh) cells. By coordinating mediated immunity cells against cancer cells, Th1 among various subsets of CD4^+^ T cells serve a direct antitumor role [13]. The N.K. cells can also directly eliminate tumor cells via several mechanisms; 1) production of cytoplasmic granules, granzymes, and perforin, 2) induction of death receptor-mediated apoptosis, or 3) secretion of tumor necrosis factor-alpha (TNF-$\alpha$) to achieve the antitumor effect through antibody-dependent cellular cytotoxicity from the expression of CD16 [13].

Macrophages play a critical role in innate immunity against cancer by preventing the accumulation of apoptotic cancer cells, which could trigger an autoimmune response. During the ideal phase, tumor cells express specific molecules on their surface that are recognized by macrophages, leading to the phagocytosis of tumor cells [13]. Macrophages can be divided into two major populations of alveolar and interstitial macrophages, where the former is more prevalent. The lung's inner surface contains a significant proportion of immune cells, with alveolar macrophages comprising 55% of these cells. These macrophages can be classified into two types, M1 and M2, based on their characteristics [10]. They can transform into different subsets in response to various stimuli. The IFN-γ factor activates macrophages, inducing them to release nitric oxide (NO) and be exposed to reactive oxygen species and lysosomal enzymes, leading to classical macrophage activation. Initially, the Th1 cells introduced the primary pathway for macrophage activation, which led to the activation of M1 macrophages. Macrophages at rest and activated by IL-4 and IL-13 are M2 macrophages, alternatively activated macrophages (AAMs), or anti-inflammatory macrophages. M2 macrophages are responsible for counteracting the effects of M1 macrophages, achieved through the secretion of IL-10. M2 macrophages also promote tissue repair by adopting an anti-inflammatory profile mediated by the TGF-β factor and other factors. This process is essential for wound healing [14]. Dendritic cells, B-cells, and macrophages are characterized as professional antigen-presenting cells (APCs). Anti-inflammatory macrophages dominate the tumor microenvironment with an impressive immune suppression function by secreted cytokines, especially transforming growth factor (TGF)-β and IL-10 [10].

Lung cancer immunotherapy provides a complex treatment additional to chemoradiotherapy by developing more comprehensive knowledge on the disturbance of antitumor immune response and the evasion mechanism of host antitumor immune defense [10, 12]. Cancer immunotherapy has priority over chemotherapy or radiotherapy for its low-risk ratio and long-lasting activity. Identifying predictive markers for predicting antitumor risks, clinical effects, and survival benefits before immunotherapies is one of the most promising directions for future research in cancer immunotherapy [13].

Scientists worldwide have developed different mathematical models for tumor disease dynamics and its characteristics. $\ddot{O}$ zk $\ddot{o}$ se et al. [15] developed a fractional-order model of the tumor-immune system using Caputo derivatives to investigate changes in the population of macrophages, active macrophages, tumor cells, and host cells. The authors in [16] developed a mathematical model to study the impact of CD4 + T cells on tumor regression, which included interactions between CD4 + cells, cytokines, tumor cells, and host cells with treatment. Kumar et al. [17] investigated the role of vitamin intervention in enhancing the immune system using a tumor-immune-vitamin (TIV) model with arbitrary order operators of Caputo-Fabrizio (C.F.) derivative and conformable fractional derivative in the Liouville-Caputo (L.C.) sense. In a research paper, Cherraf et al. [18] proposed an interaction tumor-immune model in the presence of immune chemotherapy. In their model, immune cells were recruited with a constant time delay to demonstrate the role of time delay in the stimulated accumulations of cancer cells surrounded by immune cells. Their numerical simulation suggested tumor load reduction after a few months of immuno-chemotherapy. Another tumor-immune model was both numerically and theoretically investigated by Ahmad et al. [19] for both non-singular and singular fractal fractional operators. In a chaotic and comparative study, the dynamic behavior of tumor and effector immune cells was interpreted by Kumar et al. [20] through the analysis of a fractional tumor-immune model. To explore the effect of immune checkpoints on tumor regression, Yu and Jang [21] examined mathematical models of tumor-immune interactions among CD4^+^ T cells, malignant tumor cells, and antitumor cytokine with an immune checkpoint inhibitor of CTLA-4. Dai and Liu [22] tackled an optimal control problem for a broad range of reaction–diffusion tumor-immune models with immuno-chemotherapy. The objective was to decrease the tumor cell burden while minimizing treatment costs and side effects. Fractional calculus is a branch of classical calculus concerned with integer-order formalism, which is presently used for different modeling approaches in various scientific fields of biomathematics, applied mathematics, physics, computer science, etc. (see [23–29]). Veeresha et al. [30] used the q-homotopy analyses transform method (q-HATM) to solve the fractional Schistosomiasis disease model. The results showed that their approach was easier to apply and more effective in finding numerical solutions for multi-dimensional differential equations arising in biological phenomena. In a study by Khan et al. [31], a fractional epidemic model was numerically simulated for the novel coronavirus in the sense of the Caputo operator using generalized Adams–Bashforth Moulton. Zafar et al. [32] expressed and investigated a fractional order model for Toxoplasmosis disease in cat and human populations. They proposed a fractional extension of the multistage differential transform method to model toxoplasmosis. Cui et al. [33] investigated the dynamics of Plasmodium using a time-delayed fractional-order Ross-Macdonald model for transmission periods of malaria and the order of its dynamic behavior. Abdullah et al. [34] solved a fractional temporal SEIR measles model composed of four-time fractional ordinary differential equations (TFODEs) in three stages. In the first stage, an approximate model was solved that linearized four TFODEs. Then, an analytical solution of each TFODE was obtained at each time step. A fractional Predictor–Corrector method was used in the third stage to solve the model. Hassani et al. [35] created an optimization algorithm that employs generalized polynomials to estimate the solution of an HIV infection model of CD4^+^ T cells.

Mathematical models can be adapted to try to estimate the complex dynamics of disease and simulate the appropriate and effective treatments for patients in personalized medicine. Mathematical models that are adaptable for processes critical in cancer biology will shed light on unknown points in the field of oncology. Mathematical models contribute significantly to understanding how immune and cancer cells interact and define tumor-immune dynamics [15]. It has been observed that the models made with fractional-order differential equations (FODEs) are more compatible with the truth and provide more advantages when compared with integer-order mathematical models [15]. Tumor tissue samples were collected from non-small cell lung cancer patients who had chemotherapy-naive. The best-fitted curve has been obtained using the real data of a lung cancer patient [15].

Mathematical modeling of the respiratory function as a response of heterogeneous tissue represents an attractive avenue toward narrowing the possibilities that should be tested before clinical trials. Feature extraction from modeling a respiratory function through a specific fractional order impedance model can be transposed to lung tumor dynamics. Furthermore, changes in the lung geometry along the levels of the respiratory tree are simulated, replacing the recurrent lung geometry for a tumorous lung with random asymmetry [36].

Recently, different algorithms have been developed for the numerical solutions of varying disease modeling systems. Ullah et al. [37] introduced a dynamic analysis of the susceptible-vaccinated-infected-recovered epidemic model based on mean-field approximation, evolutionary game approach, and fractional-order derivatives. Ullah et al. [38] deliberated an epidemic model based on control measures of lockdown, physical distancing, self-protection, quarantine, and isolation to study COVID-19 behavior. Din and Zainul Abidin [39] comprehensively analyzed the fractional-order vaccinated Hepatitis-B epidemic model with Mittag–Leffler kernels. Din et al. [40] analyzed a system of fractional order equations for Hepatitis B using Atangana–Baleanu Caputo (ABC) derivatives. The authors in [41] explored the numerical behavior of a fractional model that pertains to hepatitis B infection. The model was analyzed using integer order operators of differentiation, which incorporated non-local and non-singular kernels. Ain et al. [42] studied a disease transmission model of Middle East Lungs Coronavirus (MERS-CoV) in terms of Caputo fractional order variations. Kashyap et al. [43] introduced a fractional model to examine how the mass mortality of predators is affected by the fear response of prey in the Salton Sea. A novel mathematical model was introduced to investigate the effects of interleukin-10 and anti-PD-L1 administration on cancer [44]. Uçar et al. [45] numerically simulated and analyzed a new model to describe the behavior of cancer cells. Uçar et al. [46] designed a fractional susceptible–affected–infectious–suspended–recovered (SAIDR) model in the Atangana-Baleanu (A.B.) sense. Uçar [47] employed fractal-fractional operators to model hepatitis B outbreaks with the aid of Caputo derivatives and actual data. Uçar [48] extracted results from a detailed analysis of a powered smoking model by determination and education with non-singular derivatives. Zafar et al. [49] presented a numerical analysis of the Bazykin-Berzovskaya model with strong Allee effects. Zafar et al. [50] examined the numeric paradigm of a stochastic suicide substrate reaction model. Zafar et al. [51] evaluated the role of public health awareness programs in the spread of the Covid-19 pandemic. Zafar et al. [52] also worked on the fractional order dynamics of human papillomavirus. Another fractional-order model of toxoplasmosis was dynamically and numerically investigated in human and cat populations by Zafar et al. [32]. The dynamic behavior of tuberculosis was numerically modeled and simulated in the frame of different fractional derivatives by Zafar et al. [53]. Farman et al. [54] conducted a scientific investigation into the potential of genetically modified trees to reduce atmospheric carbon dioxide levels. The study introduced a system of fractional order differential equations to model the impact of these trees on the environment. The findings of this research provide valuable insights into the potential of genetically modified trees as a tool for mitigating climate change. Hasan et al. conducted an epidemiological analysis of the symmetry in Ebola virus transmission using the power law kernel [55]. Farman et al. [56] proposed a new fractional epidemic model to observe measles transmission dynamics with a constant proportional Caputo operator. An analysis of Covid-19 dynamical transmission l was also performed by Farman et al. with the Caputo-Fabrizio fractional derivative [57]. Tang et al. [58] considered the growth of artificial magnetic bacteria in a non-Newtonian Powell–Eyring nanofluid on a stretching curved surface using a porous medium. Tang et al. [59] structured the interactions of tumor–immune in the fractional derivative framework and focused on the qualitative analysis and dynamical behavior of tumor–immune cell interactions. Fioranelli et al. [60] proposed a mechanism to induce T-cells around tumor cells using the entanglement between spinors on graphene sheets interior and exterior of the human body.

Fractional derivatives are useful in modeling complex real-world phenomena. Xu et al. [61–64] have conducted several studies that explore the impact of time delays on the bifurcation of fractional systems. These studies include stage-structured predator–prey models, 4D neural networks, multi-delayed neural networks, and delayed BAM neural networks. Ahmad et al. [19, 65, 66] examined models that describe the interaction between tumors, the immune system, and vitamins. They also provided theoretical and numerical analyses of fractional fractal models with various kernels to understand this interaction better. Yuttanan and Syam [67, 68] have investigated numerical solutions to fractional partial differential equations using fractional-order generalized Taylor wavelets and the modified operational matrix method, respectively. Rawani and Khirsariya [69, 70] have used the Haar wavelets collocation method and the Homotopy perturbation general transform technique, respectively, to find numerical and analytic solutions to nonlinear partial one and two-dimensional integrodifferential equations of fractional order. These studies demonstrate the versatility and applicability of fractional derivatives in various research fields, highlighting their potential to provide new insights into complex systems.

Recent studies in the past decade have shown that compared to mathematical models of integer order, models composed of fractional-order differential equations are more advantageous and compatible with reality. This is because many biological systems display characteristics such as after-effects, hereditary properties, and memory that differential equations of integer order cannot fully represent. Fractional-order differential equations perform better in modeling these complex phenomena [14].

Cancer is a leading cause of death, accounting for nearly one in six deaths worldwide. Mathematical models can improve our understanding of cancer and help inform public health policies to promote healthy lifestyles. Based on the research mentioned above, the primary focus of this article is to develop a fractional tumor-immune interaction model for lung cancer (FTIIM-LC) and its numerical algorithm to capture the dynamic behaviors of the tumor-immune system.

Given the above consideration, this research article presents an optimization method with the below contributions.

- ▪ The FTIIM-LC model has been considered.
- ▪ This article proposes new basis functions, termed generalized Laguerre polynomials (GLPs), for the approximate solution of the FTIIM-LC model.
- ▪ The proposed operational matrices of GLPs are utilized to convert the FTIIM-LC model into a system of polynomial equations.
- ▪ The convergence of the introduced GLPs algorithm is proved in this paper.
- ▪ An optimization technique is designed for further efficiency improvement based on the Lagrange multipliers, and the optimal extent of unknown parameters is taken.
- ▪ In case of a low number of basis functions, meaningful solutions are obtained by the proposed method.
- ▪ A representation matrix form is formulated for the GLPs.
- ▪ New operational matrices of ordinary and fractional derivatives are evolved for these basis functions.

This paper is structured as follows. In Sect. "The fractional tumor-immune interaction model related to lung cancer", we formulate the FTIIM-LC model and present some definitions and valuable results of the Caputo operator. To discuss the main features of the proposed method, Sect. "Introducing a new basis function" is divided into three subsections of GLPs description, operational matrices of derivatives and function approximation. In Sect. "The convergence analysis" the convergence analysis is shown. In Sect. "The solution for FTIIM-LC", we implement the GLPs method to achieve the optimal solution of the FTIIM-LC model. Sect. "Numerical results and discussion" presents the numerical simulation and compares our method's results with other methods and real data. For a better understanding of the results, comparisons are also displayed as figures and tables. The main conclusions are drawn and given in Sect. "Conclusions".

## The fractional tumor-immune interaction model related to lung cancer

This section considers the FTIIM-LC model consisting of four fractional order differential equations to explore FTIIM-LC dynamics. The model includes four dependent variables, namely:

- *T*(*t*) represents the densities of tumor cells.
- *A*(*t*) represents the active macrophage cells.
- *M*(*t*) represents the macrophage cells.
- *W*(*t*) represents the normal tissue or host cells.

This study postulates that the tumor cells are malignant and investigates two distinct mechanisms: the degradation of macrophages by active macrophages and the conversion of macrophages into active macrophages. Additionally, supporting evidence indicates a competition between the tumor cells and healthy tissues for resources and physical area [15]. It is believed that there is a negative correlation between the densities of tumor cells and those of activated macrophages and normal cells, so the fractional system (2.1) satisfies positive conditions. The following system of nonlinear differential equations is used to formulate FTIIM-LC in the Caputo sense [15]:

2.1 $$\left\{\begin{array}{c}{}_{0}^{C}{D}_{t}^{{\upsilon }_{1}}T\left(t\right)={d}_{2}^{{v}_{1}}T\left(t\right)\left(1-\frac{T\left(t\right)}{{l}_{2}^{{\upsilon }_{1}}}\right)-{\theta }^{{v}_{1}}T\left(t\right)A\left(t\right)-{\alpha }_{1}^{{v}_{1}}W\left(t\right)T\left(t\right),\\ { }_{0}^{C}{D}_{t}^{{\upsilon }_{2}}A\left(t\right)={\rho }_{1}^{{v}_{2}}M\left(t\right)A\left(t\right)-{h}_{2}^{{v}_{2}}A\left(t\right), \\ { }_{0}^{C}{D}_{t}^{{\upsilon }_{3}}M\left(t\right)={d}_{1}^{{v}_{3}}M\left(t\right)\left(1-\frac{M\left(t\right)}{{l}_{1}^{3}}\right)-{\rho }_{2}^{{v}_{3}}M\left(t\right)A\left(t\right)-{h}_{1}^{{v}_{3}}M\left(t\right), \\ { }_{0}^{C}{D}_{t}^{{\upsilon }_{4}}W\left(t\right)={d}_{3}^{{v}_{4}}W\left(t\right)\left(1-\frac{W\left(t\right)}{{l}_{3}^{{\upsilon }_{4}}}\right)-{\alpha }_{2}^{{v}_{4}}W\left(t\right)T\left(t\right), \\ T\left(0\right)={T}_{0}\ge 0, A\left(0\right)={A}_{0}\ge 0, M\left(0\right)={M}_{0}\ge 0, W\left(0\right)={W}_{0}\ge 0,\end{array}\right.$$

where ${}_{0}^{C}{D}_{t}^{{\upsilon }_{i}}$, $i=1,\mathrm{2,3},4$, denotes the fractional derivatives of order $0<{\upsilon }_{i}\le 1$. The model (2.1) parameters and their biological meaning are given in Table 1.

**Table 1:** The parameters of the FTIIM-LC (2.1)

Parameter	Description
${\rho }_{1}$	The conversion rate of macrophages into active macrophages
${\rho }_{2}$	The degradation of macrophages due to active macrophages
${d}_{1}$	The growth rate of macrophage cells
${d}_{2}$	The growth rate of tumor cells
${d}_{3}$	The growth rate of normal tissue cells
${\alpha }_{1}$	The competition coefficient of normal tissue cells on tumor cells
${\alpha }_{2}$	The competition coefficient of tumor cells on normal tissue cells
${h}_{1}$	The death rate of macrophages
${h}_{2}$	The death rate of active macrophages
$\theta$	The rate of destruction of tumor cells due to the attack of active macrophages
${l}_{1}$	The carrying capacity of macrophages
${l}_{2}$	The carrying capacity of tumor cells
${l}_{3}$	The carrying capacity of normal tissue cells

In applied sciences, memory properties have broad applications for a better understanding of complex phenomena. Given their higher degree of freedom, the desired results are more attainable using fractional instead of integer derivatives. In various fields of chemistry, biology, physics, and economy, fractional differential equations are also helpful for perceiving hereditary and memory problems or processes, given their inherent properties of non-local operators. Readers are suggested to refer to [71, 72]. The Caputo fractional derivative, ${}_{0}^{C}{D}_{t}^{v}$ is as follows [27, 28]:

2.2 $${}_{0}^{C}{D}_{t}^{\upsilon }y(t)=\left\{\begin{array}{lll}\frac{1}{\Gamma \left(1-\upsilon \right)}{\int }_{0}^{t}{\left(t-s\right)}^{-\upsilon }{y}{\prime}(s)ds, & & 0<\upsilon <1,\\ \frac{dy(t)}{dt}, & & \upsilon =1.\end{array}\right.$$

The symbol $\Gamma (\cdot )$ represents the Gamma function, which is defined as $\Gamma (\varrho )={\int }_{0}^{\infty }{t}^{\varrho -1}{e}^{-t}dt,$ where $\varrho >0$. The Caputo fractional derivative, utilized in the convolution integral, introduces a memory effect. As a result, the Caputo fractional derivative in (2.2) retains the dynamics of the model over a long period by incorporating the history of y(t). The following equation is valid for any $\varpi \in {\mathbb{N}}$:

2.3 $${}_{0}^{C}{D}_{t}^{\upsilon }{t}^{\varpi }=\left\{\begin{array}{ll}\frac{\Gamma (\varpi +1)}{\Gamma (\varpi -\upsilon +1)} {t}^{\varpi -\upsilon },& \varpi =\mathrm{1,2},\dots ,\\ 0,& \varpi =0.\end{array}\right.$$

## Introducing a new basis function

In this section, we only recall some basic features of Laguerre polynomials (L.P.s) and propose a new class of GLPs basis functions. The operational matrices are formed to solve FTIIM-LC, and then function approximation is provided.

### Description of the GLPs

**Definition 3.1** (see [73] and related references) The L.P.s, ${\mathcal{L}}_{\mathrm{n}}(\mathrm{t})$, are solutions to second-order linear differential equations ${\mathrm{ty}}^{\mathrm{^{\prime}}\mathrm{^{\prime}}}+(1-\mathrm{t}){\mathrm{y}}^{\mathrm{^{\prime}}}+\mathrm{ny}=0,\mathrm{ n}\in {\mathbb{N}}$.

**Definition 3.2** (see [73] and related references The power series for L.P.s, ${\mathcal{L}}_{\mathrm{n}}(\mathrm{t})$, is represented as.

3.1 $${\mathcal{L}}_{n}(t)=\sum_{k=0}^{n}\frac{(-1{)}^{k}}{k!}\frac{(n)!}{(k!)(n-k)!}{t}^{k}.$$

The first L.P.s are given by:

$$\begin{array}{ll}{\mathcal{L}}_{0}(t)=1,& \\ {\mathcal{L}}_{1}(t)=-t+1,& \\ {\mathcal{L}}_{2}(t)=\frac{1}{2}({t}^{2}-4t+2),& \\ {\mathcal{L}}_{3}(t)=\frac{1}{6}(-{t}^{3}+9{t}^{2}-18t+6).& \end{array}$$

The given function $u(t)$ is generally approximated with the first terms $n+1$ L.P.s as:

3.2 $$u(t)\simeq {P}^{T} Q {\Psi }_{n}(t),$$

where

3.3 $$Q=\left(\begin{array}{llll}{q}_{00}& {q}_{01}& \cdots & {q}_{0n}\\ {q}_{10}& {q}_{11}& \cdots & {q}_{1n}\\ \vdots & \vdots & \ddots & \vdots \\ {q}_{n0}& {q}_{n1}& \cdots & {q}_{nn}\\ & & & \end{array}\right), {P}^{T}=[{p}_{0} {p}_{1} \dots {p}_{n}], {\Psi }_{n}(t)=[1 t {t}^{2} \dots {t}^{n}{]}^{T},$$

and

3.4 $${q}_{ij}=\left\{\begin{array}{ll}\frac{(-1{)}^{j}}{j!}\frac{(i)!}{(j!)(i-j)!},& i\ge j,\\ 0,& i<j.\end{array}\right.$$

**Definition 3.3** The GLPs, ${\mathcal{L}}_{m}(t)$, are formed with a change of variable. Correspondingly, ${t}^{i}$ is changed to ${t}^{i+{\beta }_{i}}$, $(i+{\beta }_{i}>0)$, on the L.P.s and provided by.

3.5 $${\mathcal{L}}_{m}(t)=\sum_{k=0}^{m}\frac{(-1{)}^{k}}{k!}\frac{(m)!}{(k!)(m-k)!}{t}^{k+{\beta }_{k}},$$

where ${\beta }_{k}$ refers to control parameters. Providing ${\beta }_{k}=0$, the GLPs are identical to the classical L.P.s.

The expression of $v(t)$ functions using GLPs can be expressed as:

3.6 $$v(t)={R}^{T} S {\Phi }_{m}(t),$$

where

3.7 $$S=\left(\begin{array}{lllll}{s}_{\mathrm{0,0}}& {s}_{\mathrm{0,1}}& {s}_{\mathrm{0,2}}& \cdots & {s}_{0,m}\\ {s}_{\mathrm{1,0}}& {s}_{\mathrm{1,1}}& {s}_{\mathrm{1,2}}& \cdots & {s}_{1,m}\\ {s}_{\mathrm{2,0}}& {s}_{\mathrm{2,1}}& {s}_{\mathrm{2,2}}& \cdots & {s}_{2,m}\\ \vdots & \vdots & \vdots & \cdots & \vdots \\ {s}_{m,0}& {s}_{m,1}& {s}_{m,2}& \cdots & {s}_{m,m}\\ & & & & \end{array}\right), {R}^{T}=[{r}_{0} {r}_{1} \dots {r}_{m}], {\Phi }_{m}(t)=[1 {t}^{1+{\beta }_{1}} {t}^{2+{\beta }_{2}} \dots {t}^{m+{\beta }_{m}}{]}^{T},$$

and

3.8 $${s}_{ij}=\left\{\begin{array}{ll}\frac{(-1{)}^{j}}{j!}\frac{(i)!}{(j!)(i-j)!},& i\ge j,\\ 0,& i<j,\end{array}\right.$$

where ${\beta }_{k}$, $k=\mathrm{1,2},\dots ,m$, are the control parameters.

The functions $T(t)$, $A(t)$, $M(t)$ and $W(t)$ can be outlined as matrices as follows:

3.9 $$\begin{array}{ll}T(t)\simeq {\mathcal{C}}_{1}^{T} {\mathfrak{D}}_{1} {\Phi }_{1}(t), A(t)\simeq {\mathcal{C}}_{2}^{T} {\mathfrak{D}}_{2} {\Phi }_{2}(t), M(t)\simeq {\mathcal{C}}_{3}^{T} {\mathfrak{D}}_{3} {\Phi }_{3}(t), W(t)\simeq {\mathcal{C}}_{4}^{T} {\mathfrak{D}}_{4} {\Phi }_{4}(t),& \end{array}$$

where

3.10 $$\begin{array}{ll}{\mathcal{C}}_{1}^{T}=[{c}_{0}^{1} {c}_{1}^{1}\dots {c}_{{m}_{1}}^{1}], {\mathcal{C}}_{2}^{T}=[{c}_{0}^{2} {c}_{1}^{2}\dots {c}_{{m}_{2}}^{2}], {\mathcal{C}}_{3}^{T}=[{c}_{0}^{3} {c}_{1}^{3}\dots {c}_{{m}_{3}}^{3}], {\mathcal{C}}_{4}^{T}=[{c}_{0}^{4} {c}_{1}^{4}\dots {c}_{{m}_{4}}^{4}],& \end{array}$$

3.11 $${\mathfrak{D}}_{1}=\left(\begin{array}{lllll}1& 0& 0& \cdots & 0\\ {d}_{\mathrm{1,0}}^{1}& {d}_{\mathrm{1,1}}^{1}& {d}_{\mathrm{1,2}}^{1}& \cdots & {d}_{1,{m}_{1}}^{1}\\ {d}_{\mathrm{2,0}}^{1}& {d}_{\mathrm{2,1}}^{1}& {d}_{\mathrm{2,2}}^{1}& \cdots & {d}_{2,{m}_{1}}^{1}\\ \vdots & \vdots & \vdots & \cdots & \vdots \\ {d}_{{m}_{1},0}^{1}& {d}_{{m}_{1},1}^{1}& {d}_{{m}_{1},2}^{1}& \cdots & {d}_{{m}_{1},{m}_{1}}^{1}\\ & & & & \end{array}\right), {\mathfrak{D}}_{2}=\left(\begin{array}{lllll}1& 0& 0& \cdots & 0\\ {d}_{\mathrm{1,0}}^{2}& {d}_{\mathrm{1,1}}^{2}& {d}_{\mathrm{1,2}}^{2}& \cdots & {d}_{1,{m}_{2}}^{2}\\ {d}_{\mathrm{2,0}}^{2}& {d}_{\mathrm{2,1}}^{2}& {d}_{\mathrm{2,2}}^{2}& \cdots & {d}_{2,{m}_{2}}^{2}\\ \vdots & \vdots & \vdots & \cdots & \vdots \\ {d}_{{m}_{2},0}^{2}& {d}_{{m}_{2},1}^{2}& {d}_{{m}_{2},2}^{2}& \cdots & {d}_{{m}_{2},{m}_{2}}^{2}\\ & & & & \end{array}\right),$$

3.12 $${\mathfrak{D}}_{3}=\left(\begin{array}{lllll}1& 0& 0& \cdots & 0\\ {d}_{\mathrm{1,0}}^{3}& {d}_{\mathrm{1,1}}^{3}& {d}_{\mathrm{1,2}}^{3}& \cdots & {d}_{1,{m}_{3}}^{3}\\ {d}_{\mathrm{2,0}}^{3}& {d}_{\mathrm{2,1}}^{3}& {d}_{\mathrm{2,2}}^{3}& \cdots & {d}_{2,{m}_{3}}^{3}\\ \vdots & \vdots & \vdots & \cdots & \vdots \\ {d}_{{m}_{3},0}^{3}& {d}_{{m}_{3},1}^{3}& {d}_{{m}_{3},2}^{3}& \cdots & {d}_{{m}_{3},{m}_{3}}^{3}\\ & & & & \end{array}\right), {\mathfrak{D}}_{4}=\left(\begin{array}{lllll}1& 0& 0& \cdots & 0\\ {d}_{\mathrm{1,0}}^{4}& {d}_{\mathrm{1,1}}^{4}& {d}_{\mathrm{1,2}}^{4}& \cdots & {d}_{1,{m}_{4}}^{4}\\ {d}_{\mathrm{2,0}}^{4}& {d}_{\mathrm{2,1}}^{4}& {d}_{\mathrm{2,2}}^{4}& \cdots & {d}_{2,{m}_{4}}^{4}\\ \vdots & \vdots & \vdots & \cdots & \vdots \\ {d}_{{m}_{4},0}^{4}& {d}_{{m}_{4},1}^{4}& {d}_{{m}_{4},2}^{4}& \cdots & {d}_{{m}_{4},{m}_{4}}^{4}\\ & & & & \end{array}\right),$$

3.13 $$\begin{array}{ll}{\Phi }_{1}(t)\triangleq [{\phi }_{0}^{1}(t) {\phi }_{1}^{1}(t)\dots {\phi }_{{m}_{1}}^{1}(t){]}^{T}, {\Phi }_{2}(t)\triangleq [{\phi }_{0}^{2}(t) {\phi }_{1}^{2}(t)\dots {\phi }_{{m}_{2}}^{2}(t){]}^{T},& \\ {\Phi }_{3}(t)\triangleq [{\phi }_{0}^{3}(t) {\phi }_{1}^{3}(t)\dots {\phi }_{{m}_{3}}^{3}(t){]}^{T}, {\Phi }_{4}(t)\triangleq [{\phi }_{0}^{4}(t) {\phi }_{1}^{4}(t)\dots {\phi }_{{m}_{4}}^{4}(t){]}^{T},& \\ & \end{array}$$

and

3.14 $${d}_{ij}^{k}=\left\{\begin{array}{ll}\frac{(-1{)}^{j}}{j!}\frac{(i)!}{(j!)(i-j)!},& i\ge j,\\ 0,& i<j,\end{array}\right. i=\mathrm{1,2},\dots ,{m}_{k}, j=\mathrm{0,1},\dots ,{m}_{k}, k=\mathrm{1,2},\mathrm{3,4},$$

3.15 $${\phi }_{i}^{k}(t)=\left\{\begin{array}{lll}1,& & i=0,\\ {t}^{i+{\beta }_{i}^{k}},& & i=\mathrm{1,2},\dots ,{m}_{k},\end{array}\right. k=\mathrm{1,2},\mathrm{3,4},$$

with ${\beta }_{i}^{k}$ that represent the control parameters.

### Operational matrices

The fractional derivatives of order $0<{\upsilon }_{i}\le 1$, of ${\Phi }_{i}(t)$, $i=\mathrm{1,2},\mathrm{3,4}$, can be shown by

3.16 $${}_{0}^{C}{D}_{t}^{{\upsilon }_{i}}{\Phi }_{i}(t)={\mathcal{D}}_{t}^{\left({\upsilon }_{i}\right)} {\Phi }_{i}(t), i=\mathrm{1,2},\mathrm{3,4},$$

where ${\mathcal{D}}_{t}^{\left({\upsilon }_{i}\right)}$ denote the following $({m}_{i}+1)\times ({m}_{i}+1)$ operational matrices of fractional derivatives

3.17 $${\mathcal{D}}_{t}^{\left({\upsilon }_{i}\right)}={t}^{-{\upsilon }_{i}}\left(\begin{array}{lllll}0& 0& 0& \cdots & 0\\ 0& \frac{\Gamma \left(2+{\beta }_{1}^{i}\right)}{\Gamma \left(2-{\upsilon }_{i}+{\beta }_{1}^{i}\right)}& 0& \cdots & 0\\ 0& 0& \frac{\Gamma \left(3+{\beta }_{2}^{i}\right)}{\Gamma \left(3-{\upsilon }_{i}+{\beta }_{2}^{i}\right)}& \cdots & 0\\ \vdots & \vdots & \vdots & \ddots & \vdots \\ 0& 0& 0& \cdots & \frac{\Gamma \left({m}_{i}+1+{\beta }_{{m}_{i}}^{i}\right)}{\Gamma \left({m}_{i}+1-{\upsilon }_{i}+{\beta }_{{m}_{i}}^{i}\right)}\\ & & & & \end{array}\right), i=\mathrm{1,2},\mathrm{3,4},$$

where ${\beta }_{j}^{i}$, ${m}_{i}$, and ${\upsilon }_{i}$ ($i=\mathrm{1,2},\mathrm{3,4}$, $j=\mathrm{1,2},\dots ,{m}_{i}$) respectively represent control parameters, basis function numbers, and fractional orders, with $\Gamma (\cdot )$ implying the gamma function.

### Function approximation

Suppose that $\{1, {\mathcal{L}}_{1}\left(t\right), {\mathcal{L}}_{2}\left(t\right), \dots ,{\mathcal{L}}_{{m}_{1}}(t)\}\subset {L}^{2}[0,T]$ is a set of GLPs and ${\mathrm{\daleth }}_{{m}_{1}}=$ Span $\{1, {\mathcal{L}}_{1}\left(t\right),{\mathcal{L}}_{2}\left(t\right),\dots , {\mathcal{L}}_{{m}_{1}}(t)\}$. Let $S(t)$ be an arbitrary element of ${L}^{2}[0,T]$. There is a finite-dimensional ${\mathrm{\daleth }}_{{m}_{1}}$ subspace of ${L}^{2}[0,T]$ space with a unique optimal approximation of F(t) in ${\mathrm{\daleth }}_{{m}_{1}}$, i.e. ${F}^{*}(t)$ such that

$$\forall G(t)\in {\mathrm{\daleth }}_{{m}_{1}}, \parallel F(t)-{F}^{*}(t){\parallel }_{2}\le \parallel F(t)-G(t){\parallel }_{2}.$$

Since ${F}^{*}(t)\in {\mathrm{\daleth }}_{{m}_{1}}$, then the unique ${c}_{0}^{1},{c}_{1}^{1},\dots ,{c}_{{m}_{1}}^{1}$ coefficients exist such that

$$F(t)\simeq {F}^{*}(t)={\mathcal{C}}_{1}^{T} {\mathfrak{D}}_{1} {\Phi }_{1}(t),$$

where Eqs. (3.11) and (3.13) are devoted to the respective definitions of${\mathcal{C}}_{1}^{T}=[{c}_{0}^{1} {c}_{1}^{1}\dots {c}_{{m}_{1}}^{1}]$, also ${\mathfrak{D}}_{1}$ and ${\Phi }_{1}(t)$.

Any square-integrable function $F(t)$, $t\in [\mathrm{0,1}]$, can be expressed in terms of GLPs as

$$F(t)=\sum_{m=0}^{\infty }{c}_{m}{\mathcal{L}}_{m}(t),$$

where ${\mathcal{L}}_{m}(t)={\sum }_{k=0}^{m}\frac{(-1{)}^{k}}{k!}\frac{m!}{k!(m-k)!}{t}^{k+{\beta }_{k}}$ is a GLPs.

**Theorem 3.4** *The error in* $\mathrm{F}(\mathrm{t})$ *approximation by the sum of its first* $(\mathrm{m}+1)$*-terms* *is* *limited to the absolute scale of all neglected coefficients. If*

3.18 $${F}_{m}(t)=\sum_{m=0}^{\infty }{c}_{m}{\mathcal{L}}_{m}(t),$$

then for all $F(t)$, $m$; and $t\in [\mathrm{0,1}]$, we have

3.19 $${E}_{{\mathcal{L}}_{m}}(t)=|F(t)-{F}_{m}(t)|\le M\sum_{k=m+1}^{\infty }|{c}_{k}|,$$

where $$M=\mathrm{max}\{|{\mathcal{L}}_{m}(t)|:t\in [\mathrm{0,1}]\}$$.

**Proof.** Since $|{\mathcal{L}}_{m}(t)|\le M$, in view of (3.18) and (3.19), we conclude that.

$${E}_{{\mathcal{L}}_{m}}\left(t\right)=\parallel F\left(t\right)-{F}_{m}\left(t\right)\parallel =\left|\sum_{k=0}^{\infty }{c}_{k}{\mathcal{L}}_{k}\left(t\right)-\sum_{k=m}^{\infty }{c}_{k}{\mathcal{L}}_{k}\left(t\right)\right|=\left|\sum_{k=m+1}^{\infty }{c}_{k}{\mathcal{L}}_{k}\left(t\right)\right| \le \sum_{k=m+1}^{\infty }|{c}_{k}|\underset{t\in [\mathrm{0,1}]}{\mathrm{max}}|{\mathcal{L}}_{m}(t)|\le M\sum_{k=m+1}^{\infty }|{c}_{k}|.$$

This completes the proof.

## The convergence analysis

In this section, the convergence analysis of GLPs is carried out in line with the following theorems.

**Theorem 3.5** ([74, 75]) *Let* $\mathrm{f}:[\mathrm{0,1}]\to {\mathbb{R}}$ *be a continuous function. A GLP of* ${\mathcal{L}}_{{\mathrm{m}}_{1}}(\mathrm{t})$ *will then be for each* $\mathrm{t}\in [\mathrm{0,1}]$ *and* $\upepsilon >0$ *such that*

$$|f(t)-{\mathcal{L}}_{{m}_{1}}(t)|<\epsilon .$$

**Proof.** Refer to [75].

**Theorem 3.6** *Let* $\mathrm{F}(\mathrm{t})$ *be an n-times continuously differentiable function on* $[\mathrm{0,1}]$ *and* ${\mathrm{F}}_{\mathrm{m}}(\mathrm{t})$ *be the best square approximation of* $\mathrm{F}(\mathrm{t})$ *given in Eq.* (3.18). *Then, we have*

$$\parallel F-{F}_{m}{\parallel }_{2}^{2}\le \frac{LM{A}^{m+1}}{(m+1)!},$$

where $L={\mathrm{max}}_{t\in [\mathrm{0,1}]}|{F}^{(m+1)}(t)|$, where $M=\mathrm{max}\{|{\mathcal{L}}_{m}(t)|:t\in [\mathrm{0,1}]\}$ and $A=\mathrm{max}\{1-{t}_{0},{t}_{0}\}$.

**Proof.** Using Taylor's expansion of $F(t)$, we obtain.

$$F(t)=F({t}_{0})+(t-{t}_{0})F{\prime}({t}_{0})+\cdots +\frac{(t-{t}_{0}{)}^{m}}{m!}{F}^{(m)}({t}_{0})+\frac{(t-{t}_{0}{)}^{m+1}}{(m+1)!}{F}^{(m+1)}(\xi ),$$

where ${t}_{0}\epsilon\,[0,\,1]$ and $\xi \in [{t}_{0},t]$. Assume now that

$$\overline{{F}_{m}}(t)=F({t}_{0})+(t-{t}_{0})F{\prime}({t}_{0})+\cdots +\frac{(t-{t}_{0}{)}^{m}}{m!}{F}^{(m)}({t}_{0}).$$

Then, we get

$$|F(t)-\overline{{F}_{m}}(t)|=\left|\frac{(t-{t}_{0}{)}^{m+1}}{(m+1)!}{F}^{(m+1)}(\xi )\right|.$$

Since ${F}_{m}(t)$ is the best square approximation of $F(t)$, we deduce that

$$\parallel F(t)-{F}_{m}(t){\parallel }_{2}^{2}\le \parallel F(t)-\overline{{F}_{m}}(t){\parallel }_{2}^{2},$$

where ${F}_{m}(t)$ is the best square approximation and $\overline{{F}_{m}}(t)$ is a GLP of degree $m$. This amounts to

$$\parallel F-{F}_{m}{\parallel }_{2}^{2}\le \parallel F-\overline{{F}_{m}}{\parallel }_{2}^{2}={\int }_{0}^{1}{\left|F\left(t\right)-\overline{{F}_{m}}\left(t\right)\right|}^{2}dt\le {\int }_{0}^{1}{\left(\frac{LM{A}^{m+1}}{\left(m+1\right)!}\right)}^{2}dt={\left(\frac{LM{A}^{m+1}}{\left(m+1\right)!}\right)}^{2}.$$

By taking the square root of both sides of the inequality above, we arrive at the necessary conclusion to finalize the proof.

**Theorem 3.7** *Let* $F(t)\in {C}^{m}([\mathrm{0,1}])$ *and* ${F}^{(k)}(t)$ *be the* *k-th derivative of* $F(t)$. *If* ${F}_{m}^{(k)}$, $k=\mathrm{1,2},\dots ,n$, *is the best approximation of* ${F}^{(k)}(t)$*, then*

3.20 $$\parallel F-{F}_{m}{\parallel }_{2}^{2}\le \frac{LM{A}^{m-k+1}}{(m-k+1)!}, k=\mathrm{1,2},\dots ,n,$$

where$${E}_{m,k}={F}^{(k)}(t)-{F}_{m}^{(k)}(t)$$, $$M=\mathrm{max}\{|{\mathcal{L}}_{m}(t)|:t\in [\mathrm{0,1}]\}$$ and $$L={\mathrm{max}}_{t\in [\mathrm{0,1}]}|{F}^{(m-k+1)}(t)|$$.

**Proof.** For any $F(t)\in {C}^{m}([\mathrm{0,1}])$, we have ${F}^{(k)}(t)\in {C}^{m-k}([\mathrm{0,1}])$. Given Theorem 3.6, we reach the desired result, which completes the proof.

**Theorem 3.8** *Let *$F(t)\in {C}^{m}([\mathrm{0,1}])$*. Let *$n-1<\upsilon \le n$* and *${}_{0}^{C}{D}_{t}^{\upsilon }{F}_{m}(t)$* be the best approximation of *${}_{0}^{C}{D}_{t}^{\upsilon }F(t)$*. Then*

$$\parallel {E}_{m,n}^{\upsilon }\parallel \le \frac{1}{\Gamma (n-\upsilon )}\frac{LM{A}^{m-n+1}}{(m-n+1)!},$$

where $${E}_{m,\upsilon }^{\upsilon }{=}_{0}^{C}{D}_{t}^{\upsilon }F(t){-}_{0}^{C}{D}_{t}^{\upsilon }{F}_{m}(t)$$ and $$L={\mathrm{max}}_{t\in [\mathrm{0,1}]}|{F}^{(m-n+1)}(t)|$$.

**Proof.** By the definition of Caputo derivative, we obtain.

$${}_{0}^{C}{D}_{t}^{\upsilon }F(t)=\frac{1}{\Gamma (n-\upsilon )}{\int }_{0}^{t}{F}^{(n)}(s)(t-s{)}^{-\upsilon -1+n}ds.$$

This implies that

$$\begin{array}{c}\parallel {E}_{m,n}^{\upsilon }\parallel ={\parallel }_{0}^{C}{D}_{t}^{\upsilon }F(t){-}_{0}^{C}{D}_{t}^{\upsilon }{F}_{m}(t)\parallel \\ =\Vert \frac{1}{\Gamma (n-\upsilon )}{\int }_{0}^{t}({F}^{(n)}(s)-{F}_{m}^{(n)}(s))(t-s{)}^{-\upsilon -1+n}ds\Vert \\ \le \frac{1}{\Gamma (n-\upsilon )}{\int }_{0}^{t}\Vert ({F}^{(n)}(s)-{F}_{m}^{(n)}(s))\Vert (t-s{)}^{-\upsilon -1+n}ds\\ \le \parallel {E}_{m,n}\parallel \\ \le \frac{1}{\Gamma (n-\upsilon )}\frac{LM{A}^{m-n+1}}{(m-n+1)!}.\end{array}$$

This completes the proof.

Now, we investigate the convergence of our method in one dimension by the following theorem.

**Theorem 3.9** *Let* $Z$ *be a normed linear space,* ${z}_{0}\in Z$*, and* $\{{x}_{n}{\}}_{n\in {\mathbb{N}}}\subset Z$ *such that* $Span \{{x}_{n}:n\in {\mathbb{N}}\}$ *is a dense subset of* $Z$. If $\{{z}_{n}{\}}_{n\in {\mathbb{N}}}\subset Z$ *is the best approximation of* ${z}_{0}$ *in* $Span \{{x}_{1},{x}_{2},{x}_{3},\dots ,{x}_{n}\}$*, then* $\{{z}_{n}{\}}_{n\in {\mathbb{N}}}\subset Z$ *converges in norm to* ${z}_{0}$.

**Proof.** By the density of $Span \{{x}_{n}:n\in {\mathbb{N}}\}$ in $Z$, there exists a sequence $\{{v}_{m}{\}}_{m\in {\mathbb{N}}}\subset Span \{{x}_{n}:n\in {\mathbb{N}}\}$ such that ${v}_{m}\to {z}_{0}$ as $m\to \infty$. We may assume that ${v}_{m}\in Span \{{x}_{1},{x}_{2},\dots ,{x}_{{n}_{m}}\}$, where ${n}_{1}<{n}_{2}<\cdots <{n}_{m}<\cdots$. In addition, from the definition of the best approximation, we obtain.

3.21 $$\parallel {z}_{{n}_{m}}-{z}_{0}\parallel \le \parallel {v}_{m}-{z}_{0}\parallel , (m\to \infty ).$$

Since the sequence $\{\parallel {z}_{n}-{z}_{0}\parallel :n\in {\mathbb{N}}\}$ is decreasing in the real numbers, by employing (3.21), we conclude that there exists a subsequence of $\{\parallel {z}_{n}-{z}_{0}\parallel :n\in {\mathbb{N}}\}$ converging to some elements of real numbers. This ensures that $\{{z}_{n}{\}}_{n\in {\mathbb{N}}}\subset Z$ converges in norm to ${z}_{0}$. This completes the proof.

**Corollary 3.10** Let $a>0$ be a fixed real number and $Z={L}^{2}([0,a])$, equipped with the norm $\parallel \cdot {\parallel }_{2}$, ${x}_{n}:={\mathcal{L}}_{n}$, the GLPs. In view of Theorem 3.9, we deduce that for each. ${z}_{0}\in {L}^{2}([0,a])$, the sequence $\{{z}_{n}{\}}_{n\in {\mathbb{N}}}$ of the best approximation of ${z}_{0}$ in $Span \{{\mathcal{L}}_{1},{\mathcal{L}}_{2},\dots ,{\mathcal{L}}_{n}\}$ converges to ${z}_{0}$ which completes the proof.

**Remark 1:** Similar to the arguments discussed in [76], we can prove that the solutions of system (2.1) are positively invariant and bounded.

## The solution for FTIIM-LC

In the present section, we will numerically solve the problem introduced in Eq. (2.1). For this purpose, the solutions $T(t)$, $A(t)$, $M(t)$ and $W(t)$ are approximated by GLPs as follows:

4.1 $$T(t)\simeq {\mathcal{C}}_{1}^{T} {\mathfrak{D}}_{1} {\Phi }_{1}(t), A(t)\simeq {\mathcal{C}}_{2}^{T} {\mathfrak{D}}_{2} {\Phi }_{2}(t), M(t)\simeq {\mathcal{C}}_{3}^{T} {\mathfrak{D}}_{3} {\Phi }_{3}(t), W(t)\simeq {\mathcal{C}}_{4}^{T} {\mathfrak{D}}_{4} {\Phi }_{4}(t),$$

where ${\Xi }^{i}=\left[{\beta }_{1}^{i} {\beta }_{2}^{i} \dots {\beta }_{{m}_{i}}^{i}\right]$, $i=\mathrm{1,2},\mathrm{3,4}$, are control parameters, and the coefficients ${\mathcal{C}}_{i}^{T}$, $i=\mathrm{1,2},\mathrm{3,4}$, are unknown. From (3.16), we have:

4.2 $$\begin{array}{c}{}_{0}^{C}{D}_{t}^{{\upsilon }_{1}}T\left(t\right)={\mathcal{C}}_{1}^{T}{\mathfrak{D}}_{1} {\mathcal{D}}_{t}^{{(\upsilon }_{1})}{\Phi }_{1}(\mathrm{t}), \\ { }_{0}^{C}{D}_{t}^{{\upsilon }_{2}}A\left(t\right)={\mathcal{C}}_{2}^{T}{\mathfrak{D}}_{2} {\mathcal{D}}_{t}^{{(\upsilon }_{2})}{\Phi }_{2}(\mathrm{t}), \\ { }_{0}^{C}{D}_{t}^{{\upsilon }_{3}}M\left(t\right)={\mathcal{C}}_{3}^{T}{\mathfrak{D}}_{3} {\mathcal{D}}_{t}^{{(\upsilon }_{3})} {\Phi }_{3}(\mathrm{t}), \\ { }_{0}^{C}{D}_{t}^{{\upsilon }_{4}}W\left(t\right)={\mathcal{C}}_{4}^{T}{\mathfrak{D}}_{4} {\mathcal{D}}_{t}^{{(\upsilon }_{4})}{\Phi }_{4}(\mathrm{t}).\end{array}$$

Regarding the initial conditions presented in (2.1), we get

4.3 $$\begin{array}{c}T\left(0\right)\simeq {\mathcal{C}}_{1}^{T} {\mathfrak{D}}_{1} {\Phi }_{1}\left(0\right), A\left(0\right)\simeq {\mathcal{C}}_{2}^{T} {\mathfrak{D}}_{2} {\Phi }_{2}\left(0\right), \\ M(0)\simeq {\mathcal{C}}_{3}^{T} {\mathfrak{D}}_{3} {\Phi }_{3}(0), W(0)\simeq {\mathcal{C}}_{4}^{T} {\mathfrak{D}}_{4} {\Phi }_{4}(0).\end{array}$$

Now, ${\mathcal{R}}_{i}(t)$ residual functions (R.F.s), $i=\mathrm{1,2},\mathrm{3,4}$, can be written for the fractional system (2.1) as:

4.4 $$\left\{\begin{array}{ll}{\mathcal{R}}_{1}(t)={\mathcal{C}}_{1}^{T} {\mathfrak{D}}_{1} {\mathcal{D}}_{t}^{\left({\upsilon }_{1}\right)} {\Phi }_{1}(t)-{d}_{2}^{{\upsilon }_{1}}{\mathcal{C}}_{1}^{T} {\mathfrak{D}}_{1} {\Phi }_{1}(t)(1-\frac{{\mathcal{C}}_{1}^{T} {\mathfrak{D}}_{1} {\Phi }_{1}(t)}{{l}_{2}^{{\upsilon }_{1}}})+{\theta }^{{\upsilon }_{1}}{\mathcal{C}}_{1}^{T} {\mathfrak{D}}_{1} {\Phi }_{1}(t){\mathcal{C}}_{2}^{T} {\mathfrak{D}}_{2} {\Phi }_{2}(t)& \\ +{\alpha }_{1}^{{\upsilon }_{1}}{\mathcal{C}}_{4}^{T} {\mathfrak{D}}_{4} {\Phi }_{4}(t){\mathcal{C}}_{1}^{T} {\mathfrak{D}}_{1} {\Phi }_{1}(t),& \\ {\mathcal{R}}_{2}\left(t\right)={\mathcal{C}}_{2}^{T} {\mathfrak{D}}_{2} {\mathcal{D}}_{t}^{\left({\upsilon }_{2}\right)} {\Phi }_{2}\left(t\right)-{\rho }_{1}^{{\upsilon }_{2}}{\mathcal{C}}_{3}^{T} {\mathfrak{D}}_{3} {\Phi }_{3}\left(t\right){\mathcal{C}}_{2}^{T} {\mathfrak{D}}_{2} {\Phi }_{2}\left(t\right)+{h}_{2}^{{\upsilon }_{2}}{\mathcal{C}}_{2}^{T} {\mathfrak{D}}_{2} {\Phi }_{2}\left(t\right), & \\ {\mathcal{R}}_{3}\left(t\right)={\mathcal{C}}_{3}^{T} {\mathfrak{D}}_{3} {\mathcal{D}}_{t}^{\left({\upsilon }_{3}\right)} {\Phi }_{3}\left(t\right)-{d}_{1}^{{\upsilon }_{3}}{\mathcal{C}}_{3}^{T} {\mathfrak{D}}_{3} {\Phi }_{3}\left(t\right)\left(1-\frac{{\mathcal{C}}_{3}^{T} {\mathfrak{D}}_{3} {\Phi }_{3}\left(t\right)}{{l}_{1}^{{\upsilon }_{3}}}\right)+{\rho }_{2}^{{\upsilon }_{3}}{\mathcal{C}}_{3}^{T} {\mathfrak{D}}_{3} {\Phi }_{3}\left(t\right){\mathcal{C}}_{2}^{T} {\mathfrak{D}}_{2} {\Phi }_{2}\left(t\right) & \\ +{h}_{1}^{{\upsilon }_{3}}{\mathcal{C}}_{3}^{T} {\mathfrak{D}}_{3} {\Phi }_{3}(t),& \\ {\mathcal{R}}_{4}(t)={\mathcal{C}}_{4}^{T} {\mathfrak{D}}_{4} {\mathcal{D}}_{t}^{\left({\upsilon }_{4}\right)} {\Phi }_{4}(t)-{d}_{3}^{{\upsilon }_{4}}{\mathcal{C}}_{4}^{T} {\mathfrak{D}}_{4} {\Phi }_{4}(t)(1-\frac{{\mathcal{C}}_{4}^{T} {\mathfrak{D}}_{4} {\Phi }_{4}(t)}{{l}_{3}^{{\upsilon }_{4}}})+{\alpha }_{2}^{{\upsilon }_{4}}{\mathcal{C}}_{4}^{T} {\mathfrak{D}}_{4} {\Phi }_{4}(t){\mathcal{C}}_{1}^{T} {\mathfrak{D}}_{1} {\Phi }_{1}(t).& \end{array}\right.$$

From Eq. (2.1), we have

4.5 $$\begin{array}{ll}{\Theta }_{1}\triangleq {\mathcal{C}}_{1}^{T} {\mathfrak{D}}_{1} {\Phi }_{1}(0)-T(0)\simeq 0, {\Theta }_{2}\triangleq {\mathcal{C}}_{2}^{T} {\mathfrak{D}}_{2} {\Phi }_{2}(0)-A(0)\simeq 0,& \\ {\Theta }_{3}\triangleq {\mathcal{C}}_{3}^{T} {\mathfrak{D}}_{3} {\Phi }_{3}(0)-M(0)\simeq 0, {\Theta }_{4}\triangleq {\mathcal{C}}_{4}^{T} {\mathfrak{D}}_{4} {\Phi }_{4}(0)-W(0)\simeq 0.& \end{array}$$

The 2-norm of the R.F.s is generated as:

4.6 $$\mathcal{Q}({\mathcal{C}}_{i},{\Xi }^{i})={\int }_{0}^{\zeta }(\sum_{j=1}^{4}{\mathcal{R}}_{j}^{2}(t))dt, i=\mathrm{1,2},\mathrm{3,4}.$$

An optimization problem is utilized to determine the unknown vectors ${\mathcal{C}}_{i}$ and ${\Xi }^{i}$, $i=\mathrm{1,2},\mathrm{3,4}$, as:

4.7 $$\mathrm{min} \mathcal{Q}({\mathcal{C}}_{i},{\Xi }^{i}), i=\mathrm{1,2},\mathrm{3,4}.$$

The optimization problem is constrained by Eqs. (4.5), with $\mathcal{Q}$ serving as the objective function. To solve this problem, it is assumed that:

4.8 $$\mathcal{J}[{\mathcal{C}}_{i},{\Xi }^{i},\xi ]=\mathcal{Q}({\mathcal{C}}_{i},{\Xi }^{i})+\xi\Theta , i=\mathrm{1,2},3,4.$$

It should be noted that $\xi$ represents the vector of Lagrange multipliers. The required and sufficient conditions can be optimally obtained by applying the Lagrange multipliers method, as outlined below:

4.9 $$\left\{\begin{array}{lll}\frac{\partial \mathcal{J}}{\partial \xi }=0,& & \\ \frac{\partial \mathcal{J}}{\partial {\mathcal{C}}_{i}}=0, i=\mathrm{1,2},\mathrm{3,4},& & \\ \frac{\partial \mathcal{J}}{\partial {\Xi }^{i}}=0, i=\mathrm{1,2},\mathrm{3,4}.& & \end{array}\right.$$

Once we have solved the system above and computed ${\mathcal{C}}_{i}$ and ${\Xi }^{i}$, $i=\mathrm{1,2},\mathrm{3,4}$, we obtain an approximate optimal solution for the problem described in Eqs. (4.1). To solve the extracted algebraic system of equations in Eq. (4.9), we utilize the "fsolve" command of Maple 18.

## Numerical results and discussion

**Fig. 1:**
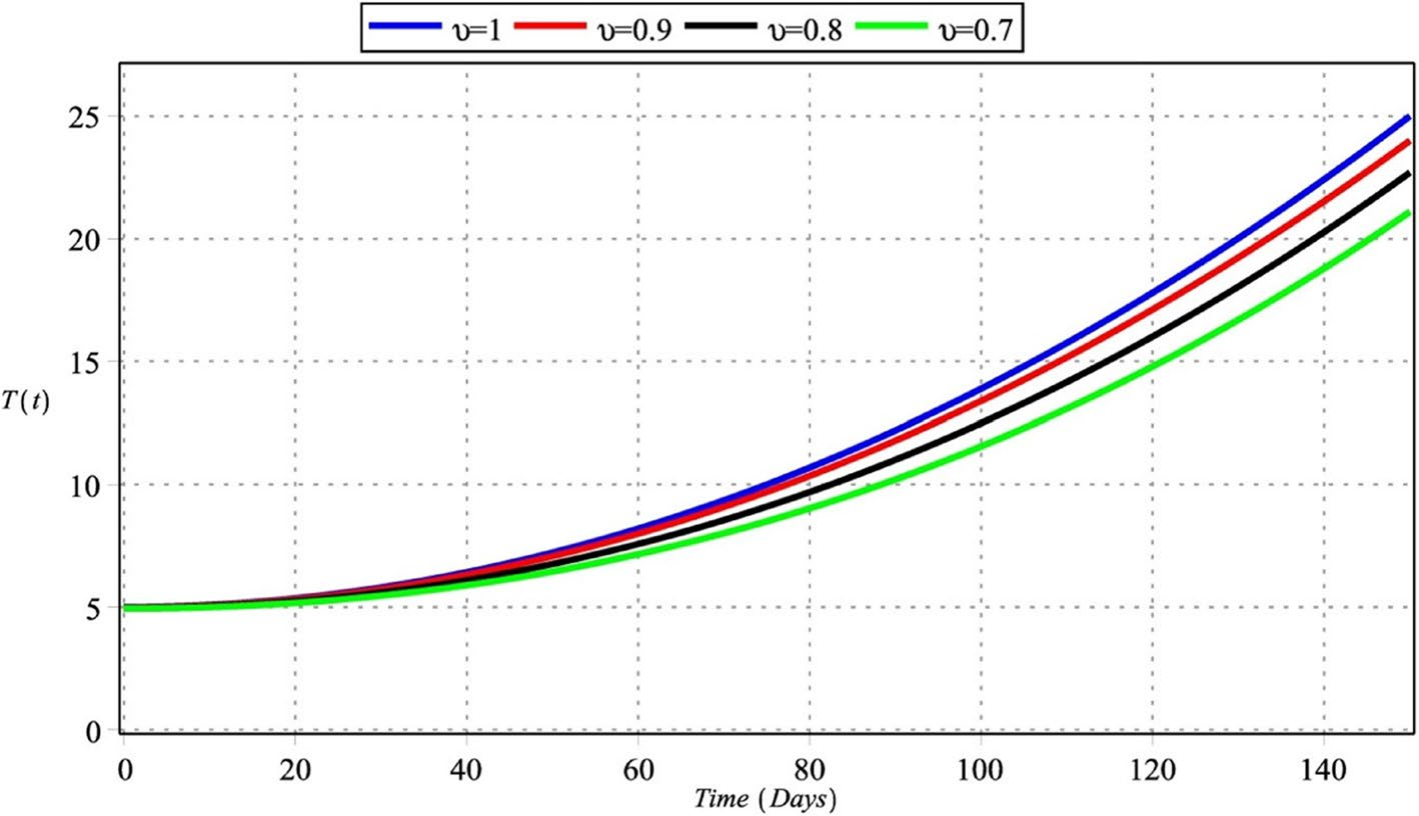
Densities of tumor cells with ${m}_{1}={m}_{2}=4$, ${m}_{3}=6$, ${m}_{4}=5$ for ${\upsilon }_{i}=\upsilon =\left\{\mathrm{0.70,0.80,0.90,1}\right\}$, $i=\mathrm{1,2},\mathrm{3,4}$

**Fig. 2:**
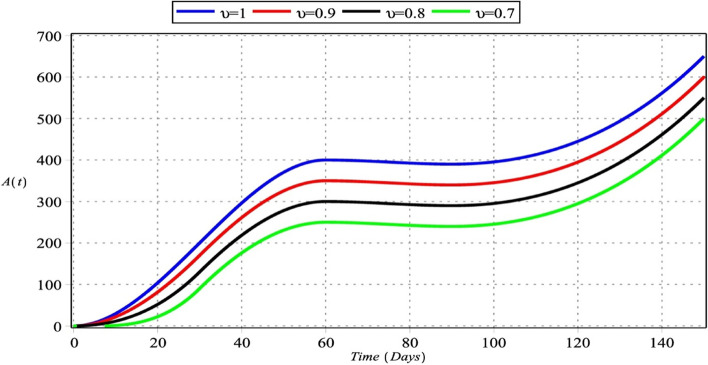
Active macrophage cells with ${m}_{1}={m}_{2}=4$, ${m}_{3}=6$, ${m}_{4}=5$ for ${\upsilon }_{i}=\upsilon =\left\{\mathrm{0.70,0.80,0.90,1}\right\}$, $i=\mathrm{1,2},\mathrm{3,4}$

**Fig. 3:**
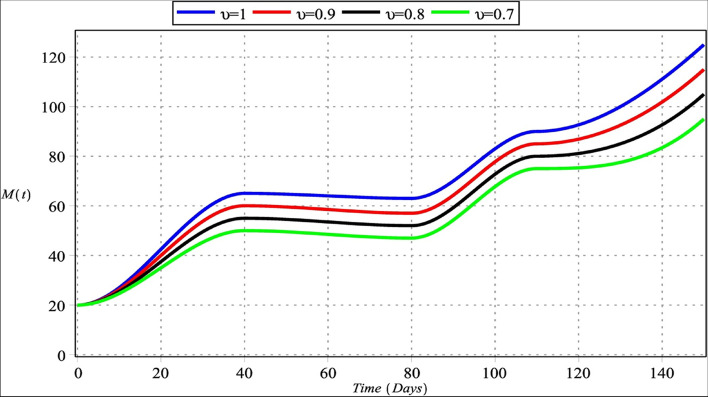
Macrophage cells with ${m}_{1}={m}_{2}=4$, ${m}_{3}=6$, ${m}_{4}=5$ for ${\upsilon }_{i}=\upsilon =\left\{\mathrm{0.70,0.80,0.90,1}\right\}$, $i=\mathrm{1,2},\mathrm{3,4}$

**Fig. 4:**
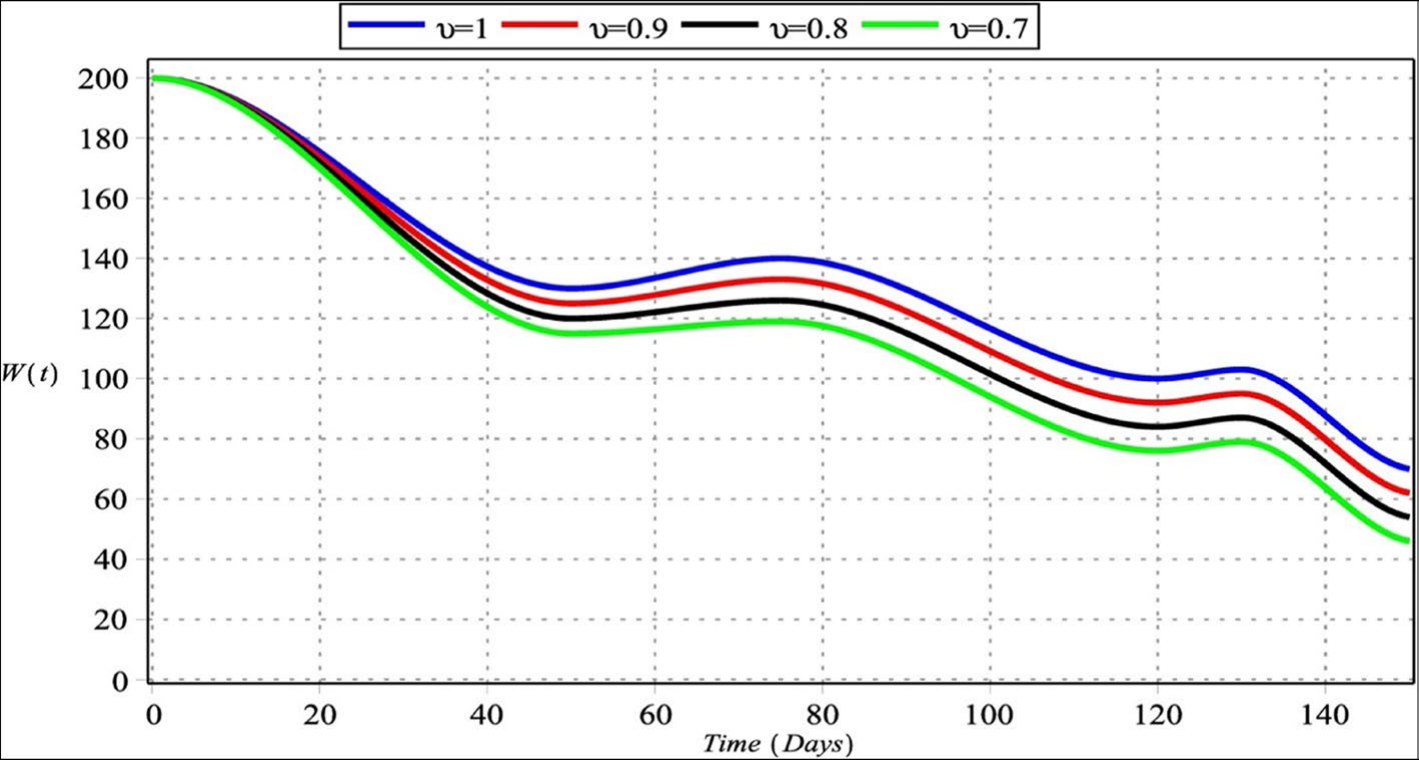
Host cells with ${m}_{1}={m}_{2}=4$, ${m}_{3}=6$, ${m}_{4}=5$ for ${\upsilon }_{i}=\upsilon =\left\{\mathrm{0.70,0.80,0.90,1}\right\}$, $i=\mathrm{1,2},\mathrm{3,4}$

**Fig. 5:**
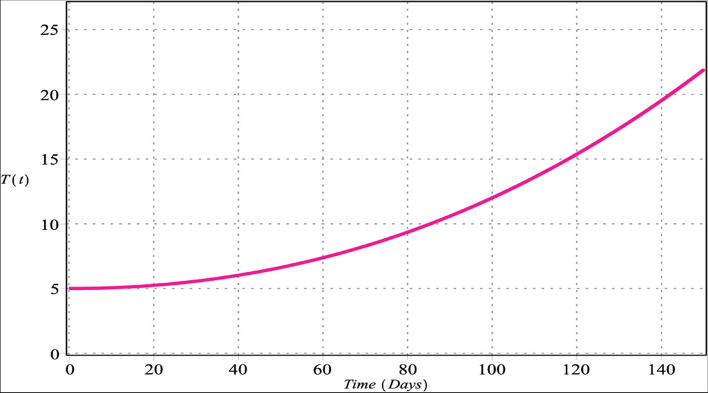
Densities of tumor cells with ${m}_{1}=3$, ${m}_{2}=5$, ${m}_{3}={m}_{4}=7$ for ${\upsilon }_{1}=0.28$, ${\upsilon }_{2}=0.43$, ${\upsilon }_{3}=0.87$, ${\upsilon }_{4}=0.96$

**Fig. 6:**
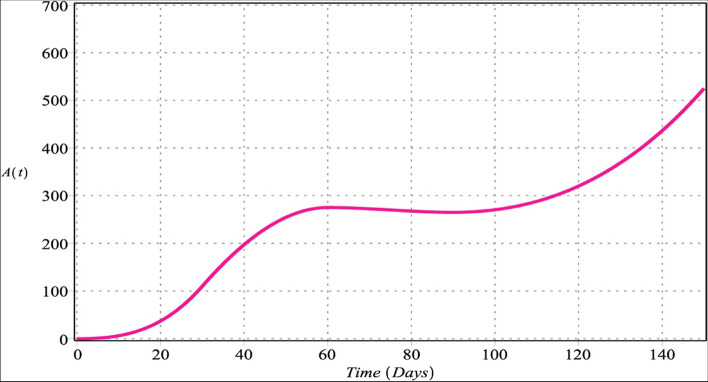
Active macrophage cells with ${m}_{1}=3$, ${m}_{2}=5$, ${m}_{3}={m}_{4}=7$ for ${\upsilon }_{1}=0.28$, ${\upsilon }_{2}=0.43$, ${\upsilon }_{3}=0.87$, ${\upsilon }_{4}=0.96$

**Fig. 7:**
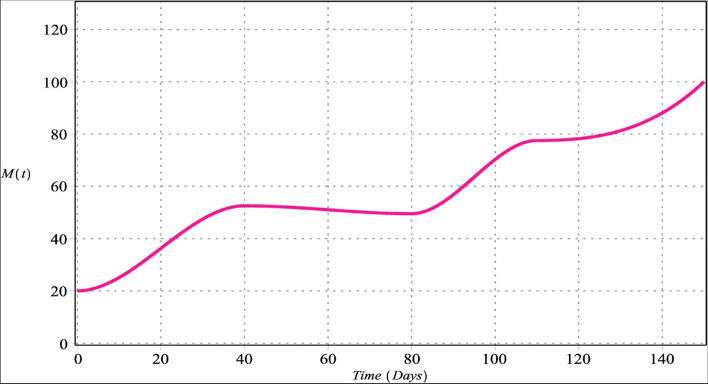
Macrophage cells with ${m}_{1}=3$, ${m}_{2}=5$, ${m}_{3}={m}_{4}=7$ for ${\upsilon }_{1}=0.28$, ${\upsilon }_{2}=0.43$, ${\upsilon }_{3}=0.87$, ${\upsilon }_{4}=0.96$

**Fig. 8:**
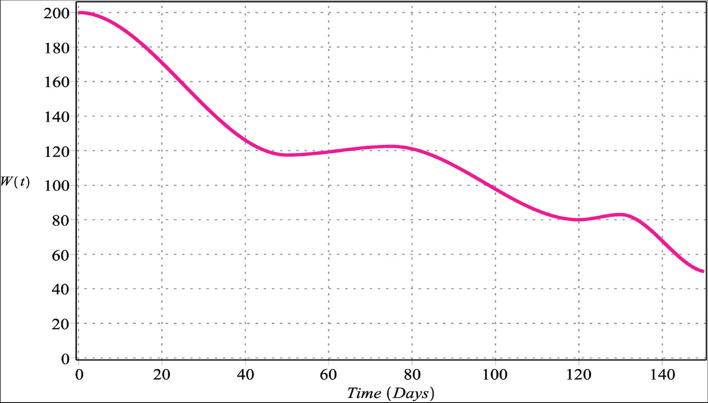
Host cells with ${m}_{1}=3$, ${m}_{2}=5$, ${m}_{3}={m}_{4}=7$ for ${\upsilon }_{1}=0.28$, ${\upsilon }_{2}=0.43$, ${\upsilon }_{3}=0.87$, ${\upsilon }_{4}=0.96$

The GLPs method is utilized for the numerical results of FTIIM-LC. Table 2 [15] provides the relevant data. The initial conditions for the simulation are $T(0)=5$, $A(0)=0$, $M(0)=20$ and $W(0)=200$. By utilizing the given parameter values, we conduct simulations for the four state variables $\{T(\mathrm{t}),A(\mathrm{t}),M(\mathrm{t}),W(\mathrm{t})\},$ as depicted in Figs. 1, 2, 3 and 4, with ${m}_{1}={m}_{2}=4$, ${m}_{3}=6$, ${m}_{4}=5$, $\zeta =150$ for ${\upsilon }_{i}=\upsilon =\left\{\mathrm{0.70,0.80,0.90,1}\right\}$, $i=\mathrm{1,2},\mathrm{3,4}$. The runtime values and the R.F.s optimal values of the proposed method are reported in Tables 3 and 4, with ${m}_{1}={m}_{2}=4$, ${m}_{3}=6$, ${m}_{4}=5$ for ${\upsilon }_{i}=\upsilon =\left\{\mathrm{0.70,0.80,0.90,1}\right\}$, $i=\mathrm{1,2},\mathrm{3,4}$. The approximate solutions are plotted in Figs. 5, 6, 7 and 8 with ${m}_{1}=3$, ${m}_{2}=5$, ${m}_{3}={m}_{4}=7$ for ${\upsilon }_{1}=0.28$, ${\upsilon }_{2}=0.43$, ${\upsilon }_{3}=0.87$, and ${\upsilon }_{4}=0.96$.

**Table 2:** The parameters of the FTIIM-LC (2.1) [15]

Parameter	Values	Unit
${l}_{1}$	$5.0785\times 1{0}^{7}$	$Cells$
${l}_{2}$	$2.7785\times 1{0}^{5}$	$Cells$
${l}_{3}$	$5.4621\times 1{0}^{6}$	$Cells$
${h}_{1}$	$4.3884\times 1{0}^{-14}$	$Da{y}^{-1}$
${h}_{2}$	$0.8809$	$Da{y}^{-1}$
${\alpha }_{1}$	$4.3930\times 1{0}^{-14}$	$(Cell Day{)}^{-1}$
${\alpha }_{2}$	$0.7609$	$(Cell Day{)}^{-1}$
${d}_{1}$	$0.9000$	$Da{y}^{-1}$
${d}_{2}$	$0.5045$	$Da{y}^{-1}$
${d}_{3}$	$0.6169$	$Da{y}^{-1}$
${\theta }_{2}$	$0.0140$	$(Cell Day{)}^{-1}$
${\rho }_{1}$	$0.0937$	$(Cell Day{)}^{-1}$
${\rho }_{2}$	$0.0122$	$(Cell Day{)}^{-1}$

**Table 3:** The proposed method runtime (in seconds) for different choices of ${m}_{i}$, $i=\mathrm{1,2},\mathrm{3,4}$

				CPU time	CPU time	CPU time	CPU time
${m}_{1}$	${m}_{2}$	${m}_{3}$	${m}_{4}$	$\upsilon =0.70$	$\upsilon =0.80$	$\upsilon =0.90$	$\upsilon =1$
4	4	6	5	$34.12$	$35.76$	$37.41$	$35.29$

**Table 4:** The optimal values of R.F.s with different choices of ${m}_{i}$, $i=\mathrm{1,2},\mathrm{3,4}$

				RF	RF	RF	RF
${m}_{1}$	${m}_{2}$	${m}_{3}$	${m}_{4}$	$\upsilon =0.70$	$\upsilon =0.80$	$\upsilon =0.90$	$\upsilon =1$
4	4	6	5	$7.2589\mathrm{E}-09$	$6.9641\mathrm{E}-09$	$4.9276\mathrm{E}-09$	$1.5394\mathrm{E}-09$

Figures 1 and 5 show that the densities of tumor cells are constantly increasing. The density of tumor cells is a crucial factor in drug resistance and metastasis regulation. Cancer cell density grows with cell proliferation in a space bounded by the basement membrane and enclosed by the stromal matrix [77]. There is ample evidence of the evolutionary development of tumor cells from somatic ones arising from the synergy of genetic damage accumulation, genetic feature variation, and specific environmental factor effects. During tumor progression, cellular invasion and metastasis are correlated with cell-to-cell communication and signaling [78].

Figures 2 and 6 show that the number of activated macrophages is increasing. Figures 3 and 7 show that the number of macrophages is also rising. Macrophages play different roles ranging from their antitumor activity in the early stages of cancer development to their tumor-promoting function in established cancer [79]. Infiltration of tumor-associated macrophages is recruited to the tumor site and associated with lung tumor stage, metastasis focus, and unfavorable prognosis in solid tumors [80–82]. Macrophages comprise most immune infiltration in tumors and have significantly different effects on tumorigenesis depending on their phenotype within the tumor microenvironment (TME) [83].

Figures 4 and 8 show a gradually decreasing number of normal host cells. The tumor stroma comprises vasculature, extracellular matrix, basement membrane, immune cells, and fibroblasts. Despite tumor-suppressing properties of stroma host cells, they are changing with malignancy and instigating tumor cell invasion, growth, and metastasis. The progression and development of cancer strongly depend upon interactions among stromal and tumor cells [84].

**Remark 2:** From a numerical standpoint, our approach is distinct from other spectral methods in various aspects. The goal is to minimize the difference between the numerical and exact solutions. Spectral methods, including Legendre, Lagrange, Jacobi, and Chebyshev polynomials, require the determination of coefficients to express the solution of a differential equation as a set of basis functions. The determination of coefficients can be achieved using three common techniques: collocation, tau, and Galerkin. In this case, the residual process and the residual 2-norm are utilized to convert the research problem into an optimization problem, yielding unknown optimal parameters. As a result, optimality conditions are established for a nonlinear system of algebraic equations with undetermined coefficients.

On the other hand, arbitrary smooth functions can be approximated using singular Sturm–Liouville eigenfunctions of Jacobi, Chebyshev, Lagrange, Hermite, or Legendre polynomials. However, these basis functions are not optimal for approximating non-analytic functions since the rate at which the number of basis functions approaches infinity is slower than the truncation error converging to zero in the approximation. Therefore, Generalized Laguerre Polynomials (GLPs) may be more effective alternatives.

## Conclusions

This paper presents an optimization approach based on GLPs combined with Lagrange multipliers for analyzing FTIIM-LC. The model's outcomes align with actual data, indicating a steady increase in tumor cell, macrophage, and activated macrophage densities and a gradual decrease in normal host cells. The proposed scheme was tested, and the results were presented in graphical and tabular forms. The computations demonstrate the method's accuracy, even in cases with limited basis functions. The article concludes that the algorithm's results help clarify FTIIM-LC's biological behavior and justify theoretical statements. Also, the approach's adaptability makes it helpful in exploring various domains in medicine and biology. The authors suggest that the methodology's versatility enables researchers to address nonlinear partial differential equations, such as fractional Klein-Gordon, fractional diffusion wave, fractional telegraph, and fractional optimal control problems. Future research could focus on applying the proposed method to these other models and investigating their theoretical and practical implications.

Additionally, the proposed method could be applied to other types of cancer, such as breast or prostate cancer, to investigate the dynamics of tumor-immune interactions in these cases. This could involve developing new models for these types of cancer or adapting the existing FTIIM-LC model to suit the specific characteristics of the tumor in question. Also, the proposed method could be further refined and improved by incorporating additional factors that influence tumor-immune interactions, such as the role of cytokines or chemokines in the tumor microenvironment. This could lead to more accurate predictions of disease progression and treatment outcomes.

## Data Availability

All data generated or analyzed during this study are included in this published article.

## Abbreviations

APCs: Antigen-presenting cells
SCLC: Small-cell lung carcinoma (NSCLC) non-small cell lung carcinoma
FTIIM-LC: Fractional tumor-immune interaction model related to lung cancer
CTLs: Cytotoxic T lymphocytes
GLPs: Generalized Laguerre polynomials
L.P.s: Laguerre polynomials
